# Applying Harmonic Optical Microscopy for Spatial Alignment of Atrial Collagen Fibers

**DOI:** 10.1371/journal.pone.0013917

**Published:** 2010-11-09

**Authors:** Yu-Wei Chiu, Men Tzung Lo, Ming-Rung Tsai, Yi-Chung Chang, Rong-Bin Hsu, Hsu-Yu Yu, Chi-Kuang Sun, Yi-Lwun Ho

**Affiliations:** 1 Division of Cardiology, Department of Internal Medicine, Far-Eastern Memorial Hospital, Taipei, Taiwan; 2 Research Center for Adaptive Data Analysis, National Central University, Taoyuan, Taiwan; 3 Department of Electrical Engineering, Graduate Institute of Photonics and Optoelectronics, National Taiwan University, Taipei, Taiwan; 4 Graduate Institute of Communication Engineering, National Taiwan University, Taipei, Taiwan; 5 Department of Surgery, National Taiwan University Hospital and National Taiwan University College of Medicine, Taipei, Taiwan; 6 Graduate Institute of Clinical Medicine, National Taiwan University Hospital and National Taiwan University College of Medicine, Taipei, Taiwan; 7 Division of Cardiology, Department of Internal Medicine, National Taiwan University Hospital and National Taiwan University College of Medicine, Taipei, Taiwan; CNRS, France

## Abstract

**Background:**

Atrial fibrosis creates a vulnerable tissue for atrial fibrillation (AF), but the spatial disarray of collagen fibers underlying atrial fibrosis is not fully elucidated.

**Objective:**

This study hypothesizes that harmonics optical microscopy can illuminate the spatial mal-alignment of collagen fibers in AF via a layer-by-layer approach.

**Patients and Methods:**

Atrial tissues taken from patients who underwent open-heart surgery were examined by harmonics optical microscopy. Using the two-dimensional Fourier transformation method, a spectral-energy description of image texture was constituted and its entropy was used to quantify the mal-alignment of collagen fibers. The amount of collagen fiber was derived from its area ratio to total atrial tissue in each image. Serum C-terminal pro-collagen pro-peptide (CICP), pro-matrix metalloproteinase-1 (pro-MMP-1), and tissue inhibitor of matrix metalloproteinase-1 (TIMP-1) were also evaluated.

**Results:**

46 patients were evaluated, including 20 with normal sinus rhythm and 26 with AF. The entropy of spectral-energy distribution of collagen alignment was significantly higher in AF than that in sinus rhythm (3.97±0.33 vs. 2.80±0.18, *p*<0.005). This difference was more significant in the permanent AF group. The amount of collagen was also significantly higher in AF patients (0.39±0.13 vs. 0.18±0.06, *p*<0.005) but serum markers of cardiac fibrosis were not significantly different between the two groups.

**Conclusions:**

Harmonics optical microscopy can quantify the spatial mal-alignment of collagen fibers in AF. The entropy of spectral-energy distribution of collagen alignment is a potential tool for research in atrial remodeling.

## Introduction

Atrial fibrosis creates a vulnerable tissue for atrial fibrillation (AF) [1]. The increase in extracellular matrix protein enlarges the intercellular space and causes delayed electric signal transduction between cells, which increases the possibility of AF [1], [2]. Boyden el al. found that increased amounts of connective tissue between cardiomyocytes have high susceptibility for initiating AF [3], [4]. AF maintenance is promoted by an atrial vulnerable tissue that is suitable for the initiation and continuation of the re-entering wavelets [5]. Accumulating fibrillar and non-fibrillar collagen leads to excessive atrial fibrosis and maintenance of AF [6]. In AF patients, increased serum degenerating products of collagen I, collagen III, and fibronectin all imply changes of collagen fibers [7]–[9]. However, the spatial disarray of collagen fibers underlying atrial fibrosis is not fully elucidated.

Harmonics-based optical microscopy is widely applied in biomedical research [10]–[12]. Confocal microscopy can also quantify collagen fibers but the tissues need to be stained and are destroyed. In reflectance mode confocal microscopy, staining is not needed but higher power is required to achieve better S/N (signal-to-noise) ratio. Therefore the tissue will be damaged in some degree [13]. Non-fluorescence-based SHG microscopy uses infrared excitation wavelengths that minimize the energy deposition, decrease the tissue damage, and increase the tissue penetration while maintaining intrinsically high spatial resolution without staining. Using SHG, collagen fibers of atrial tissue can be observed directly without staining and had good axial and lateral resolution [14]–[17]. Different layers of atrial tissue can also be observed sequentially to construct three-dimensional images.

This study hypothesizes that harmonics optical microscopy can illuminate the spatial mal-alignment of collagen fiber in AF through a layer-by-layer approach. The aims are: 1) observe collagen fibers in atrial tissues by harmonics optical microscopy; 2) quantify the spatial disarray of collagen fibers by mathematical methods; and 3) compare differences of collagen alignment and serum fibrosis markers in patients with and in those without AF.

## Materials and Methods

### Patients

Patients undergoing open-heart surgery for coronary artery disease or valvular heart disease were enrolled and those with either AF or normal sinus rhythm were recorded. AF was considered paroxysmal when it was self-terminating in <24 hours and persistent when it was documented on sequential 12-lead electrocardiograms without any intervening periods of sinus rhythm for at least 3 months preceding enrollment. Permanent AF was defined as sustained arrhythmia despite cardioversion.

The exclusion criteria included: 1. patients with cardiogenic shock (systolic pressure <80 mmHg for more than 30 min, or inotropic agents or intra-aortic balloon counter-pulsation needed); 2. patients who had undergone major surgery within the past 6 weeks; 3. patients with concomitant infection; and 4. patients with abnormal liver function and hepatitis B or C virus infection (GPT>100 IU/L or total bilirubin >2 mg/dl). Around 1×1 cm^2^ of tissue was taken from the right lateral atrium during open-heart surgery in all patients and incubated directly into optimal cutting temperature (O.C.T) compound (Miles Inc., Elkhart, NH, USS). The samples were stored at −20°C and defrosted at room temperature before harmonics optical microscopy. The Ethics Committee of National Taiwan University Hospital approved the study and all patients provided written informed consent.

### Histology

Formalin-fixed, paraffin-embedded atrial tissue sections of 5 mm thickness were de-paraffinized in xylol and a descending alcohol sequence (i.e., 100%, 96%, and 75%) and then brought into distilled water. These sections were stained with Masson's trichrome.

### Laser-scanning, higher-harmonic optical microscopy of atrial tissue

**Figure 1:**
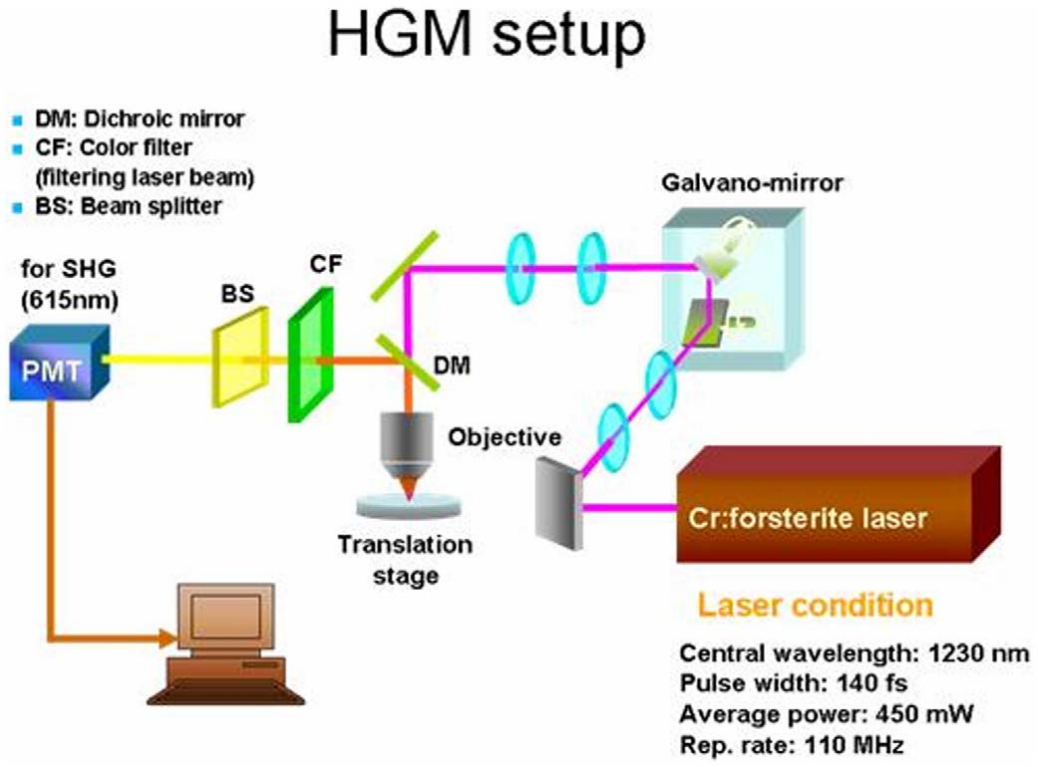
The laser-scanning, higher-harmonic optical microscope system. The system was adapted from an Olympus FV300 scanning unit combined with an Olympus BX51 upright microscope. The atrial tissue was placed on the translation stage without fixation and stain. BS, beam splitter; CF, color filter; DM, dichroic mirror; PMT, photo-multiplier tube.

This system was adapted from an Olympus FV300 scanning unit combined with an Olympus BX51 upright microscope. All optics were modified to allow the passage of 1200–1350 nm infrared light (Fig. 1). The light source was a home-built Cr:forsterite laser, functioning at 1230 nm with a repetition rate of 110 MHz and a pulse width of 140 fs, a repetition rate of 110-MHz, and 450-mW average output. All optics were modified to allow the passage of the excitation source light. We adapted the high-speed galvanometer mirrors (GM) inside the FV300 scanning system with a BX51 upright microscope and a high NA objective (LUMPlanFl/IR 60X/water/NA 0.90), all from Olympus, Real-time 2D scanning was accomplished with a pair of high-speed galvanometer mirrors inside the scanning unit. A collimated laser beam was coupled into the scanning system connecting to an Olympus BX51 microscope with an aperture-fitting tube lens [18].

In order to visualize the entire atrial structures, the working distance of the objective had to be longer than 1.5 mm. Through a 2-mm-working-distance high numerical-aperture (NA) infrared objective (LUMPlanF1/IR 60X/water/NA0.9, Olympus), the excitation laser pulse was focused onto the desired location inside the specimen and scanned with a spot size close to its diffraction limit. The average power after the objective was ∼100 mW. The backward-propagating optical harmonics signals were collected with a high-NA oil-immersion condenser. The signals were guided into two photo-multiplier tubes (PMT), which were synchronized with galvanometer mirrors and used to respectively record the interference-filtered SHG signals point-by-point to form 2D sectioned images.

**Figure 2:**
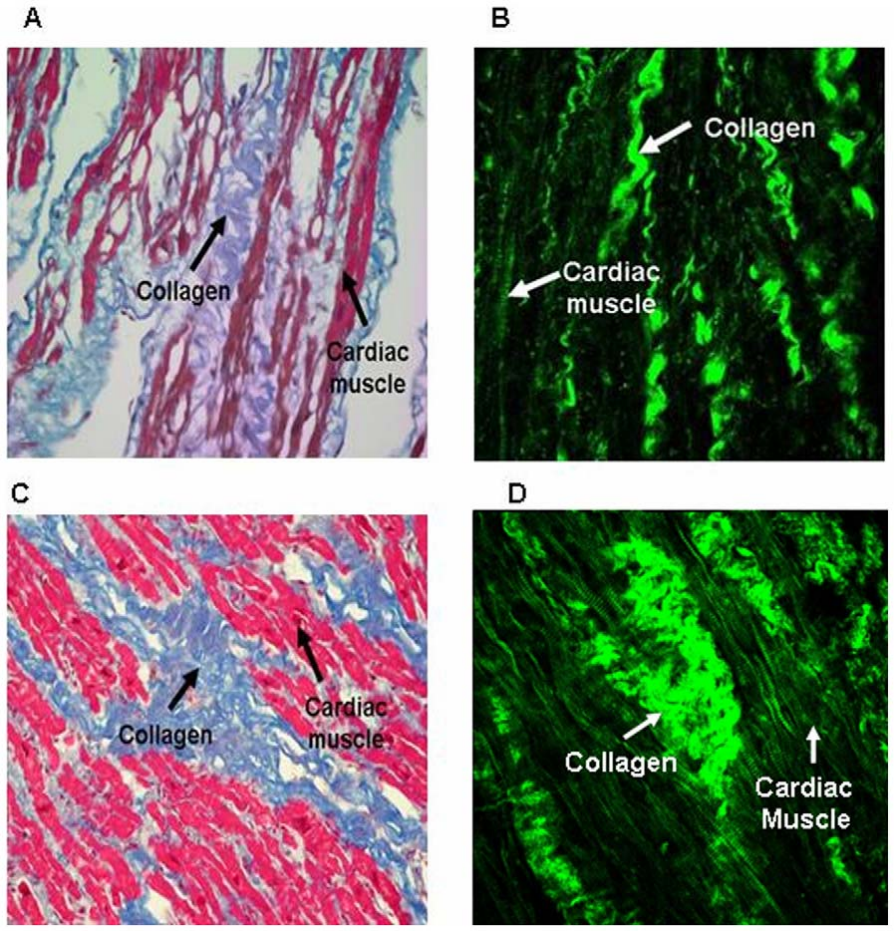
The traditional stain image and second harmonic generation image of atrial tissue. (**A**) The Masson's trichrome stain image (400×) of atrial tissue in a patient with sinus rhythm. (**B**) The second harmonic generation (SHG) image of the same atrium tissue. (**C**) The Masson's trichrome stain image (400×) of atrial tissue in a patient with atrial fibrillation (AF). (**D**) The SHG image of the same atrial tissue.

In addition, stereographic three-dimensional images of the entire atrium were obtained by controlling the depth of focus inside the specimens and by continuously scanning the same specimens with a recording speed of 0.25–2.5 sec per frame, with 512×512 scanning points. The deepest penetration depth was around 500 um but the image quality is not so good. The images we used for analysis were within the penetration depth of 200µm. During the observation, the atrium was kept at room temperature (20±1°C). The harmonics signals collected were represented by pseudo-colors, with green for SHG (Fig. 2). The finest spatial resolutions for the SHG modalities inside the atrium were experimentally measured to be 400–500 nm and 500–600 nm in lateral and on the order of 1.3 um in axial.

### Texture analysis of atrium SHG images

**Figure 3:**
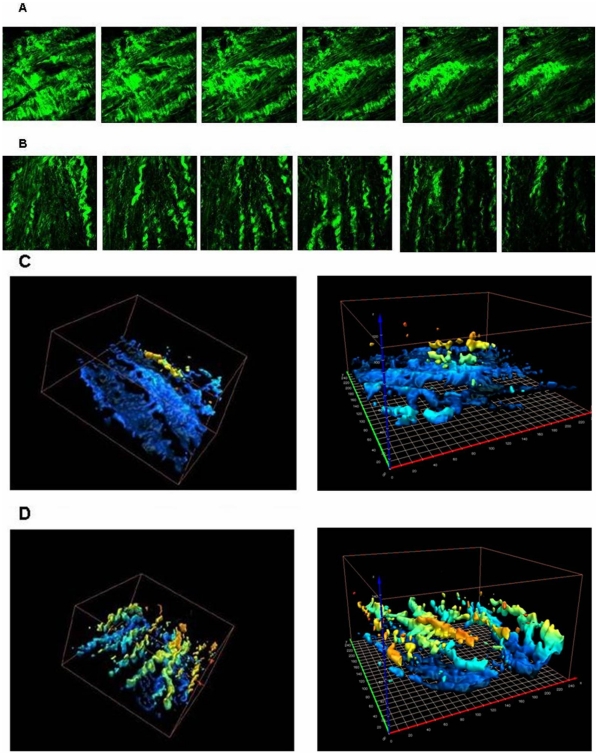
Sequential imaging and three dimensional reconstruction of SHG image. (**A**) Sequential imaging of SHG image in the depth separation of 3.9µm of each image in a patient with AF. (**B**) Sequential imaging of SHG image in the depth separation of 3.9µm of each image in a patient with NSR. (**C**) Three-dimensional reconstruction of SHG images in (A). Each square equals 10×10 µm2. (**D**) Three-dimensional reconstruction of SHG images in (B). Each square equals 10×10 µm2.

**Figure 4:**
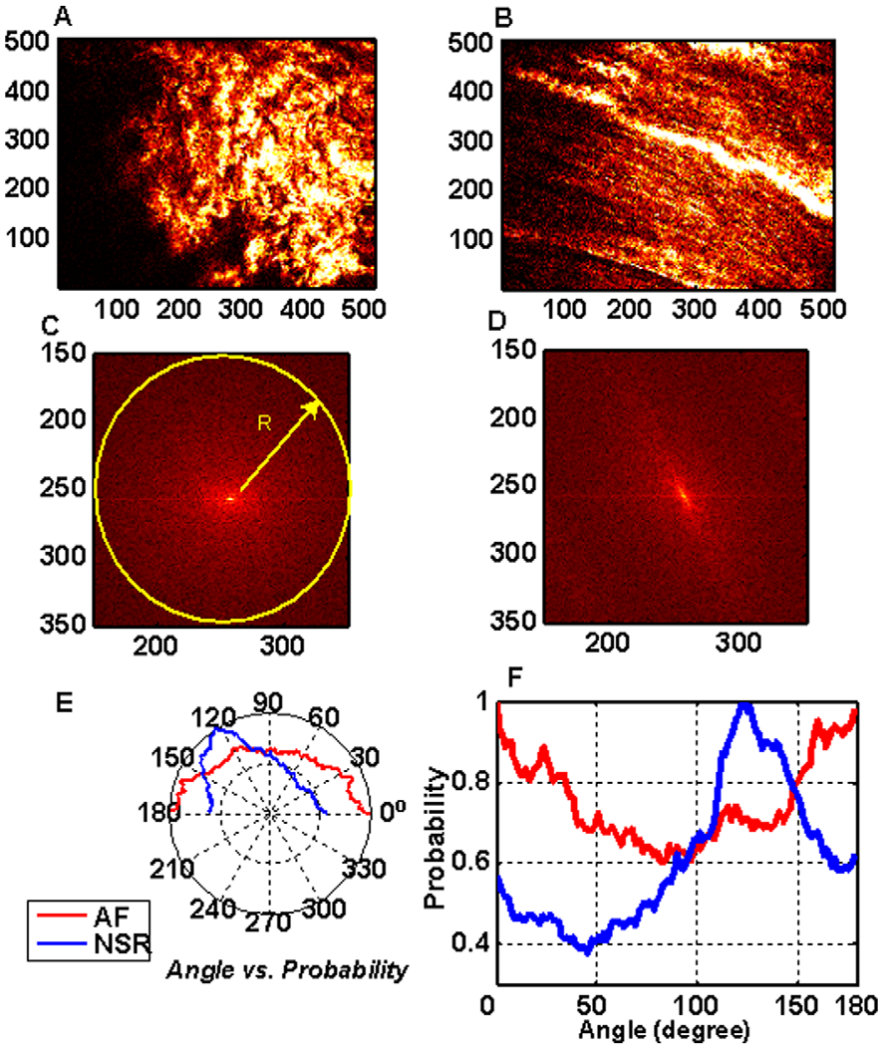
Texture analysis of atrium SHG image. (**A**) The second harmonic generation (SHG) image of atrium in a patient with atrial fibrillation (AF). (**B**) The SHG image of the atrium in a patient with normal sinus rhythm (NSR). (**C**) Two-dimensional Fourier transformation for the alignment of collagen fibers in (A). (**D**) Two-dimensional Fourier transformation for the alignment of collagen fibers in (B). (**E**) The polar plots of integrated frequency power over angle for alignment of collagen fibers in patients with either AF or NSR. (**F**) The function of integrated frequency power of angle for alignment of collagen fibers in patients with either AF or NSR.

All of the three-dimensional pictures were separated into continuous two-dimensional images (Fig. 3A, 3B). Three-dimensional reconstruction of these images was done to create the spatiality model of collagen alignment (Fig 3C, 3D). For each specimen, 25 sequential 2D images were used for texture analysis. Figures 4A and 4B provide examples of SHG images acquired from patients with either AF or sinus rhythm. Apparently, the collagens and cardiomyocytes in patients with sinus rhythm were in regular alignment, whereas they were twisted together in AF patients. To confirm the visual inspection, the spectral approach based on two-dimensional Fourier transformation was applied to describe the pattern of texture of harmonic images. The reason we chose Fourier analysis as the representation scheme is that the Fourier analysis is ideally suited for to detect the globally regular pattern in an image by identifying the highly concentrated energy in the spectrum, which generally are quite difficult to detect with other texture analysis utilized in spatial domain because of the local nature of these techniques [19]. As a result, Fourier analysis is an important image processing tool that has also been used to determine the orientation and anisotropy of the microstructure, such as collagen fibers [20]–[22]. Many studies have combined SHG microscopy and a Fourier transform to analyze the orientation or periodicity of the studied biological structures, such as skeletal muscle [23], corneal tissues [24], and collagen gels [25].

Let 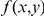 be an spatial function, representing the image intensity at point 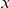, and 
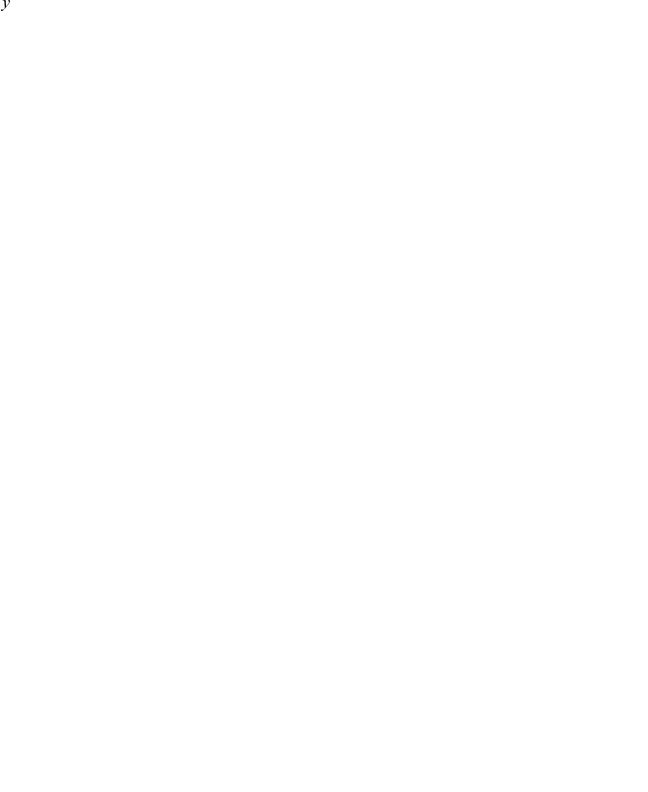
, while its spatial Fourier transform is defined by
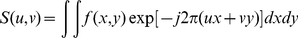
(1)


Where 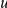 and 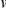 indicate the spatial frequency variables along 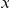 and 
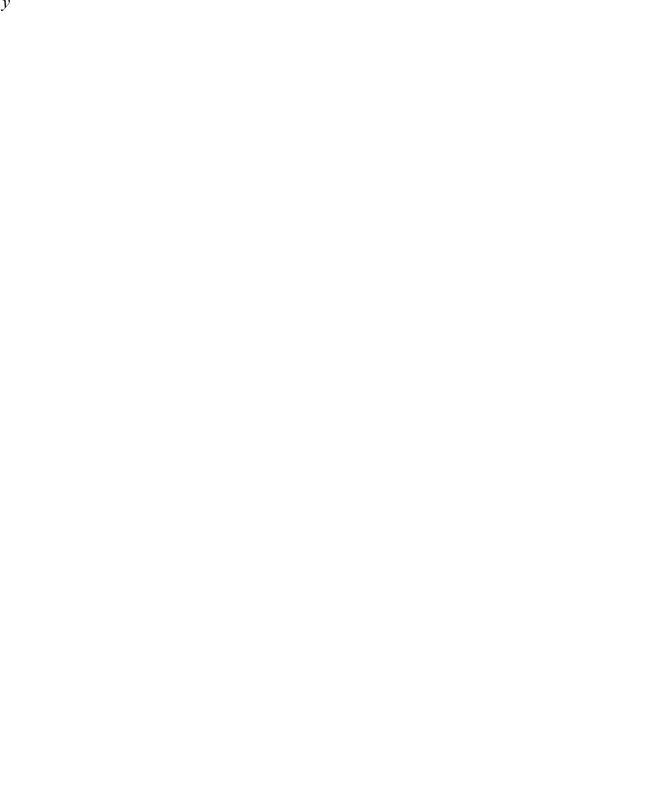
, respectively. The directional or anisotropic texture presents the quasi-periodic pattern in spatial domain, and thus exhibits the concentrated power along a straight line in the two-dimensional spectrum. In contrast, the random textures usually corresponded to the isotropic power distribution in spatial frequency domain [19]. Consequently, a disk-shaped pattern was observed in the two-dimensional spectrum for twisted SHG textures in AF (Fig 4C). Nevertheless, the power was very concentrated over certain directions in the spectral domain for regular SHG textures in sinus rhythm (Fig. 4D). To further quantify the homogeneity of texture direction, the two-dimensional Fourier spectrum was then first expressed in polar coordinates denoted as 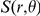, where S is the spectrum function and r and θ correspond to parameters of angle and distance in the polar coordinate system, respectively. By integrating 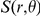 over r,
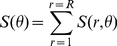
(2)We can obtain the global description of how the energy distributed with texture direction. Where R denotes the radius of a circle, and R is chosen as half of the minimum value between image length and width.

Figs. 4E and 4F show the polar and original plots of 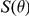 for patients with either AF or sinus rhythm. The intensity of 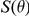 was clearly almost uniformly distributed over 0°∼180° for AF patients. This indicated the feature of isotropic textures. In contrast, it showed predominance in 110°∼140° directions for patients with sinus rhythm. The function of 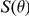 divided by its integral over 0∼π, denoted as 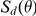 behaves as the general probability distribution. Thus, based on the information theory, we can utilize the Shannon entropy,
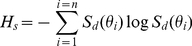
(3)to measure the randomness of the texture directions (i.e., the quantitative value of the amount of texture directions which the image contains) [26]. That is, higher 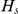 values meant that the textures in the spatial domain were more isotropic or random. Accordingly, 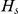, the entropy of the spectral-energy description was used to quantify the spatial mal-alignment of collagen fibers.

Moreover, since the SHG amplitudes of collagen were significantly greater than those of cardiomyocytes, the SHG images were separated into 3 different regions that corresponded to collagen, cardiomyocytes, and background by the multi-level luminance threshold method [27]. The areas of these regions were then estimated and the ratio of collagen was calculated as:
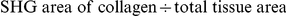



This ratio was used to define the extent of collagen in the atrium.

### Laboratory analysis

Serum samples (20 ml) were obtained from each patient and stored at −60°C until analysis. The concentration of matrix degradation products in the serum was determined from serum C-terminal collagen propeptide (CICP), pro-matrix metalloproteinase-1 (pro-MMP-1), and tissue inhibitor of matrix metalloproteinase-1 (TIMP-1) levels. Commercially available enzyme-linked immuno-assay kits (Quantikine Human TIMP-1, R&D Systems, USA; Quantikine Human pro-MMP-1, R&D Systems, USA) measured serum TIMP-1 and pro-MMP-1 levels, while serum CICP level was determined by a commercially available sandwich enzyme immuno-array kit (METRA CICP kit, QUIDEL, USA). The intra- and inter-assay variations were each <5%. Personnel blinded to the patient's clinical data performed measurements.

### Statistical analysis

All continuous results were expressed as mean ± standard deviation (SD). Comparisons between groups for continuous data were made using the Mann-Whitney U test or Student's *t* test. Multivariable linear regression analyses were conducted for significant association between variables on univariate analyses. A *p* value <0.05 was considered statistically significant.

## Results

### Patients

Baseline clinical and demographic features of the study population are shown in Table 1. 46 patients were enrolled, including 20 with normal sinus rhythm and 26 with AF. Among the 26 AF patients, 8 had paroxysmal, 8 had persistent, and 10 had permanent AF. There were no significant differences in gender, age, cardiovascular risk factors, left ventricular ejection fraction, and use of angiotensin-converting enzyme inhibitors or statins between the AF and sinus rhythm groups. Patients with AF had a larger left atrium diameter and used more amiodarone.

**Table 1:** Basic characteristics.

	AF	Sinus rhythm	P value
Number	26	20	
Age (yrs)	64±15	59±19	0.25
Gender (M/F)	15/11	11/9	0.43
VHD (No)	22	16	0.22
Hypertension (No)	18	15	0.34
Diabetes Mellitus (No)	12	10	0.40
Hyperlipidemia (No)	10	9	0.33
Smoking (No)	11	9	0.43
LVEF (%)	62.0±14.2	64.0±13.3	0.31
Left Atrium (mm)	53.1±8.0	42.6±5.7	<0.005
Statin	8	6	0.48
Amiodarone	16	0	<0.005
β blocker	20	16	0.40
ACE inhibitors	21	16	0.48
Spironolactone	5	3	0.36
Calcium channel blocker	7	6	0.41

Abbreviations: AF, Atrial fibrillation; VHD, valvular heart disease; LVEF, left ventricular ejection fraction; ACE, angiotensin converting enzyme.

### Masson's trichrome staining and SHG images

The Masson's trichrome staining and SHG images of atrial tissues were compared. The cardiomyocytes and collagen fibers were demonstrated clearly by the SHG imaging technique. In the atrial tissue of the sinus rhythm group, the alignment of collagen network was regular (Figs. 2A and 2B). In AF patients, the cardiomyocytes were surrounded by thick collagen fibers that separated cells from each other. Tissues from AF patients revealed larger inter-cellular spaces and increased extracellular matrix components (Figs. 2C and 2D), with fragmented cardiomyocytes and distorted collagen fibers.

### Entropy analysis and collagen amount analysis

558 images in sinus rhythm patients and 661 images in AF patients, including 198 from paroxysmal, 202 from persistent and 261 from permanent AF patient, were used for analysis. We counted the average entropy and collagen area ratio of each patient and did the statistic analysis then. In AF patients, the entropy of collagen alignment was higher than in patients with sinus rhythm (3.97±0.33 vs. 2.80±0.18, *p*<0.005) (Table 2). Patients with permanent AF had higher isotropic entropy data than those with either persistent or paroxysmal AF (3.98±0.21 vs. 3.70±0.51, p = 0.049; 3.98±0.21 vs. 3.68±0.30, *p* = 0.047) (Table 3). The collagen area ratio was significantly higher in AF patients than that in sinus rhythm subjects (0.39±0.13 vs. 0.18±0.06; *p*<0.005).

**Table 2:** Findings of harmonic optical microscopy and serum levels of type 1 collagen turnover products and.

	AF (N = 26)	Sinus rhythm (N = 20)	P value
Entropy of collagen alignment	3.97±0.33	2.80±0.18	<0.005
Area Ratio of collagen	0.39±0.13	0.18±0.06	<0.005
CICP (ng/ml)	70.22±28.35	58.43±22.37	0.09
Pro-MMP-1 (ng/ml)	0.64±0.54	0.78±0.48	0.20
TIMP-1 (ng/ml)	139.51±82.78	104.29±45.24	0.06

Abbreviations: CICP, C-terminal propeptide of collagen type-1; pro-MMP-1, pro-matrix metalloproteinase-1; TIMP-1, Tissue inhibitor of matrix metalloproteinase-1.

**Table 3:** Entropy of collagen alignment in different types of AF.

	Paroxysmal AF(N = 8)	Persistent AF(N = 8)	Permanent AF(N = 10)
Entropy of collagen alignment	3.68±0.30	3.70±0.51	3.98±0.21

*p* = 0.047 permanent AF vs. paroxysmal AF.

*p* = 0.049 permanent AF vs. persistent AF.

*p* = NS paroxysmal AF vs. persistent AF.

### Serum markers of collagen turnover

The findings are summarized in Table 2. Serum CICP and TIMP-1 levels tended to be higher in the AF group compared with those in the sinus group, but did not reach statistical significance. Serum pro-MMP-1 tended to be lower in the AF group but also had no statistically significant difference. The correlation coefficients of serum collagen turnover markers and entropy of collagen alignment were not significant (Table 4).

**Table 4:** Correlation coefficient of collagen angle entropy and serum markers.

	TIMP-1	CICP	Pro-MMP-1
Entropy of collagen alignment	0.391	0.117	−0.221
P value	NS	NS	NS

## Discussion

Atrial remodeling has a crucial role in AF [1]. Electrical remodeling, such as shortening of the effective refractory period and loss of rate adaptation, cannot explain the whole course of AF [28]. Structural remodeling is the so-called “second factor” that facilitates the maintenance of AF [5] and includes atrial enlargement, cardiomyocyte de-differentiation, and cellular degeneration with fibrosis, and is found in both human and animal models. Cardiomyocytes in normal myocardial tissue are electrically coupled primarily in an end-to-end fashion by intercellular gap-junctional complexes. Reactive fibrosis results in extracellular matrix expansion between myocyte bundles, while reparative fibrosis replaces degenerating myocytes. Both patterns of collagen distribution are exaggerated during structural remodeling [1].

This study demonstrated that SHG images derived from harmonics optical microscopy can quantify the amount and spatial mal-alignment of atrial collagen fibers in AF. Harmonic optical microscopy allows for the observation of fresh atrial tissue directly without staining and for obtaining sequential images by scanning the whole depth of the material. Collagen fiber has a highly crystalline triple-helix structure that is not centro-symmetric. Thus SHG microscopy is an ideal tool to observe collagen fiber structure [14]–[17]. Due to its nonlinearity, SHG is sensitive to collagen orientation disorder and can be used to analysis the arrangement of collagen. Traditional staining image of collagen is often difficult to interpret and analyze; in contrast the collagen in SHG image had better resolution, better contrast to other tissue, and made image analysis more easily. In this research, we revealed and quantified the arrangement of the collagen by using SHG microscopy. The thick ex-vivo tissue such as our atrium specimen requires backward rather than forward imaging detection. The SHG images were obtained from direct backward generation and backscattering of forward SHG signals. Unlike muscle tissue in which the SHG was dominated by backscattering of forward SHG, that of collagen fibers was dominated by the backward SHG [29]. Three-dimensional reconstruction of these images is feasible and can further illuminate the structural remodeling of atrial tissues, while good resolution of collagen and atrial cardiomyocytes provide more accuracy for area ratio calculation by the image analyzer. Clinical implication for SHG imaging for heart tissue in vivo is not technically available now. If we would like to observe the cardiomyocytes directly, we have to insert the laser catheter into the heart chamber by cardiac catheter technique. The collecting light signals from backward direction is disturbed by circulating red blood cells in the heart chamber and the motion effect of heart, resulting in the deterioration of image quality. Therefore, real-time observation via intra-cardiac laser catheter for SHG signal is not technically feasible at present. The potential in-vivo application of harmonic optical microscopy still needs further research. But in ex-vivo conditions, it is a good modality for further assessment of microstructure of myocardium. We could dissect the whole picture via better resolution and analyze the signal more precisely through this new platform.

The two-dimensional Fourier transformation method characterizes spatial variations of images. The more regular the texture is, the more concentrated the energy-spectral distribution derived from the 2D Fourier transformation of analyzed image will be. Accordingly, the irregularity of collagen fiber distribution can be quantified by estimating the homogeneity of frequency intensity over directional vectors. The intra -individual analysis for the images taken from same patient was also performed. There was some variation in the collagen array layer by layer, but no variation in entropy of collagen alignment (p = 0.45). This study also demonstrates that collagen alignment is markedly disoriented in atrial tissue in AF patients. Moreover, the increasing collagen area ratio implies the expansion of collagen fibers and the replacement of cardiomyocytes in the atrium. Patients with permanent AF have more significant collagen mal-alignment than those with persistent and paroxysmal AF. The aforementioned results suggest a potential role of SHG images derived from harmonics optical microscopy in the research of atrial remodeling, and the application of Fourier transform-based techniques of image analysis is a new modality to investigate changes in the collagen alignment in atrium.

Through Western blot and immuno-histochemical analysis, atrial tissues in AF are characterized by alterations in collagen I and III synthesis/degeneration [6], [30]–[34]. Few studies have reported that serum levels of collagen turnover products are elevated in patients with AF [35], [36]. However, serum levels of collagen turnover products were not significantly different between patients with and those without AF in this study. The discrepancy between the present and previous studies [8], [9], [35], [36] could be explained by different systolic function and concomitant medications. Ventricular fibrosis in heart failure further enhanced and concomitant medications (such as angiotensin converting enzyme inhibitors and statin) suppressed the extracellular matrix turnover [37]–[39]. In previous studies comparing patients with AF and sinus rhythm, the mean ejection fraction of left ventricle was lower than that in our study. Few of them had mentioned about the detailed medication in their patients. In contrast, our patients had higher ejection fraction (above 60%) and most of them had used angiotensin converting enzyme inhibitors and statin (more than 80%) in either sinus rhythm or AF group. These phenomena may be the reasons of the less difference of the serum collagen turnover markers in our studies. These serum markers do not correlate with the entropy of collagen alignment, and SHG data rather than serum markers offered more information on atrial remodeling in AF. The increasing collagen area ratio and entropy of collagen alignment found by SHG data also support the hypothesis that fibrosis progresses from paroxysmal AF to permanent AF. Therefore, our study suggests that SHG microscopy can serve potentially as a non-destroyed and reproducible tool for collagen fibers imaging and as an early diagnostic marker for AF.

There are several study limitations in our study. First, only the right lateral atrium was used for analysis. Nonetheless, the extracellular fibrosis was consistent in different portions of the left and right atrium in a pig rapid-pacing AF model [32]. Thus, the right lateral atrium tissue may be representative of changes of the whole atrium. Second, the case numbers in this study were small. Large-scale studies are warranted to confirm the clinical applications of this new technique for AF. Third, the atrium tissue was taken from patients submitted to open-heart surgery. Therefore the SHG images and serum collagen turnover markers level may be differed in healthy humans.

In conclusion, harmonics optical microscopy is an ideal tool to analyze the arrangement of collagen fibers and can quantify the spatial mal-alignment of collagen fibers in AF by Fourier transform-based techniques. The three-dimensional reconstruction spatiality model and entropy of spectral-energy distribution of collagen alignment are potential tools in the research of atrial remodeling.

## Supporting Information

File S1 Dynamic 3D model of atrial collagen fibers in AF patients in Fig 3(c).
(3.63 MB MPG)

File S2 Dynamic 3D model of atrial collagen fibers in NSR patient in Fig 3(d).
(4.40 MB MPG)
